# Scaling behaviours of deep learning and linear algorithms for the prediction of stroke severity

**DOI:** 10.1093/braincomms/fcae007

**Published:** 2024-01-10

**Authors:** Anthony Bourached, Anna K Bonkhoff, Markus D Schirmer, Robert W Regenhardt, Martin Bretzner, Sungmin Hong, Adrian V Dalca, Anne-Katrin Giese, Stefan Winzeck, Christina Jern, Arne G Lindgren, Jane Maguire, Ona Wu, John Rhee, Eyal Y Kimchi, Natalia S Rost

**Affiliations:** J. Philip Kistler Stroke Research Center, Department of Neurology, Massachusetts General Hospital, Harvard Medical School, Boston, MA 02114, USA; UCL Queen Square Institute of Neurology, University College London, London WC1N 3BG, UK; J. Philip Kistler Stroke Research Center, Department of Neurology, Massachusetts General Hospital, Harvard Medical School, Boston, MA 02114, USA; J. Philip Kistler Stroke Research Center, Department of Neurology, Massachusetts General Hospital, Harvard Medical School, Boston, MA 02114, USA; J. Philip Kistler Stroke Research Center, Department of Neurology, Massachusetts General Hospital, Harvard Medical School, Boston, MA 02114, USA; J. Philip Kistler Stroke Research Center, Department of Neurology, Massachusetts General Hospital, Harvard Medical School, Boston, MA 02114, USA; University of Lille, Inserm, CHU Lille, U1171—LilNCog (JPARC)—Lille Neurosciences & Cognition, Lille F-59000, France; J. Philip Kistler Stroke Research Center, Department of Neurology, Massachusetts General Hospital, Harvard Medical School, Boston, MA 02114, USA; Computer Science and Artificial Intelligence Lab, Massachusetts Institute of Technology, Cambridge, MA 02139, USA; Athinoula A. Martinos Center for Biomedical Imaging, Department of Radiology, Massachusetts General Hospital, Charlestown, MA 02129, USA; Department of Neurology, University Medical Center Hamburg-Eppendorf, Hamburg 20251, Germany; Athinoula A. Martinos Center for Biomedical Imaging, Department of Radiology, Massachusetts General Hospital, Charlestown, MA 02129, USA; Department of Computing, Imperial College London, London SW7 2RH, UK; Institute of Biomedicine, Department of Laboratory Medicine, Sahlgrenska Academy, University of Gothenburg, Gothenburg 41390, Sweden; Department of Clinical Genetics and Genomics Gothenburg, Region Västra Götaland, Sahlgrenska University Hospital, Gothenburg 41345, Sweden; Department of Neurology, Skåne University Hospital, Lund 22185, Sweden; Department of Clinical Sciences Lund, Neurology, Lund University, Lund 22185, Sweden; University of Technology Sydney, Ultimo, NSW 2007, Australia; Athinoula A. Martinos Center for Biomedical Imaging, Department of Radiology, Massachusetts General Hospital, Charlestown, MA 02129, USA; Department of Neurology, Massachusetts General Hospital, Boston, MA 02139, USA; Department of Neurology, Feinberg School of Medicine, Northwestern University, Evaston, IL 60201, USA; J. Philip Kistler Stroke Research Center, Department of Neurology, Massachusetts General Hospital, Harvard Medical School, Boston, MA 02114, USA

**Keywords:** ischaemic stroke, stroke severity, prediction, deep learning, scaling behaviour

## Abstract

Deep learning has allowed for remarkable progress in many medical scenarios. Deep learning prediction models often require 10^5^–10^7^ examples. It is currently unknown whether deep learning can also enhance predictions of symptoms post-stroke in real-world samples of stroke patients that are often several magnitudes smaller. Such stroke outcome predictions however could be particularly instrumental in guiding acute clinical and rehabilitation care decisions. We here compared the capacities of classically used linear and novel deep learning algorithms in their prediction of stroke severity. Our analyses relied on a total of 1430 patients assembled from the MRI-Genetics Interface Exploration collaboration and a Massachusetts General Hospital–based study. The outcome of interest was National Institutes of Health Stroke Scale–based stroke severity in the acute phase after ischaemic stroke onset, which we predict by means of MRI-derived lesion location. We automatically derived lesion segmentations from diffusion-weighted clinical MRI scans, performed spatial normalization and included a principal component analysis step, retaining 95% of the variance of the original data. We then repeatedly separated a train, validation and test set to investigate the effects of sample size; we subsampled the train set to 100, 300 and 900 and trained the algorithms to predict the stroke severity score for each sample size with regularized linear regression and an eight-layered neural network. We selected hyperparameters on the validation set. We evaluated model performance based on the explained variance (*R*^2^) in the test set. While linear regression performed significantly better for a sample size of 100 patients, deep learning started to significantly outperform linear regression when trained on 900 patients. Average prediction performance improved by ∼20% when increasing the sample size 9× [maximum for 100 patients: 0.279 ± 0.005 (*R*^2^, 95% confidence interval), 900 patients: 0.337 ± 0.006]. In summary, for sample sizes of 900 patients, deep learning showed a higher prediction performance than typically employed linear methods. These findings suggest the existence of non-linear relationships between lesion location and stroke severity that can be utilized for an improved prediction performance for larger sample sizes.

## Teaser

Bourached *et al*. contrast linear and deep learning–based algorithms in their prediction performances of stroke severity depending on the training set sample sizes. They find that linear regression outperforms deep learning–based algorithms for smaller training samples comprising lesion location information of 100 patients, while deep learning excels in the case of larger samples (*N* = 900).

## Graphical Abstract

**Graphical Abstract:**
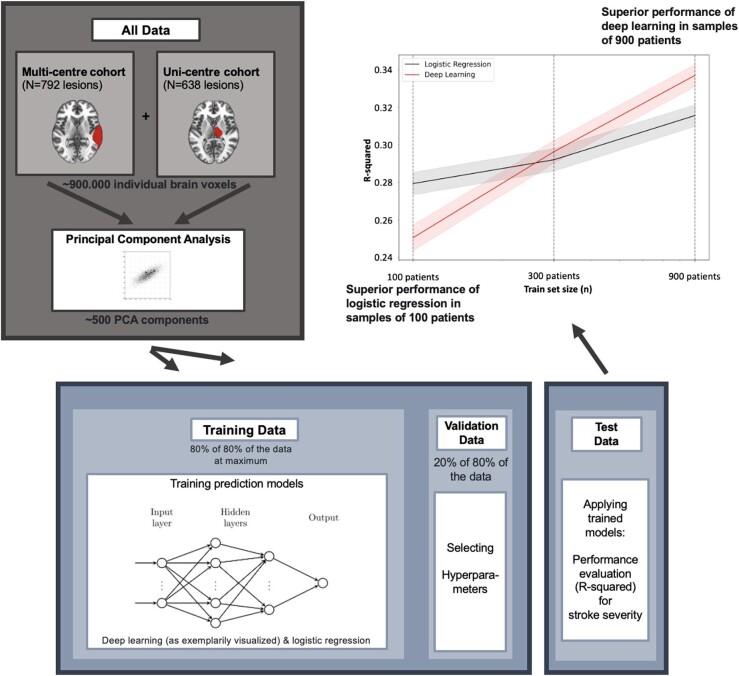

## Introduction

Recent estimates suggest that ∼12 million people experienced a new stroke worldwide in 2019, while a total of ∼100 million people lived that year after having experienced a previous stroke.^1^ Additionally, stroke is the most burdensome neurological disorder, as highlighted by evaluations of years of full health lost to disability and death.^2,3^

Thus, stroke is both a commonly occurring and a socioeconomically relevant disease which renders any efforts to optimize stroke care exceptionally important. Precision medicine has been a key focus of these efforts in recent years, as it holds promise to optimize patient outcomes. From a methodological standpoint, the realization of this individualized care is particularly linked to the fruitful combination of artificial intelligence and big data.^4^ In fact, many stroke outcome studies have employed classic machine learning algorithms to predict stroke outcomes from various sources of neuroimaging data.^5-7^ As such, there has thus been a quick adaptation of novel and powerful methods as soon as computational resources permitted their use.

The capacity of deep learning for pattern recognition and classification has been especially emphasized for complex and unstructured problems such as chemistry,^8^ physics,^9^ art history^10-14^ and even human behaviour.^15,16^ Similarly, the promises of deep learning for medicine are innumerable. Indeed, some specific biomedical fields have seen major advancements. Examples can be seen in algorithms capable of the prediction of protein structures based on their amino acid sequence (AlphaFold),^17^ histopathological evaluations of tumour tissue^18^ or enhanced automatic medical image processing, relating to preprocessing^19^ and automatic segmentation of pathological brain changes.^20-22^

However, the published literature on *deep learning*–based stroke outcome prediction is comparatively sparse. This may be due to previously relatively small available data set sizes of oftentimes only a few hundred subjects in stroke outcome studies. Two recent studies^23,24^ showed a benefit of deep learning algorithms for the prediction of favourable functional outcome post-stroke. More specifically, both teams trained convolutional neural networks (CNNs) on acute imaging data, that is, non-contrast CT data^23^ and MRI-based diffusion-weighted imaging (DWI) data.^24^ They then compared the resulting performance with established, basic clinical scores, such as the ASPECT score.^25^ Both studies relied on ∼200–300 patients in total for model derivation and validation. In contrast, Chauhan and colleagues^26^ performed a direct comparison of (non-linear) deep learning algorithms and linear algorithms and their capacities to predict language impairments post-stroke based on DWI-derived lesion location information. They did not find any evidence for a superiority of deep learning but noted that a combination of deep learning for the refinement of DWI information and ridge regression was most optimal in their specific setup. Importantly, their analyses were based on a maximum sample size of 132 patients.

There have been substantial data set size increases in stroke ‘neuroimaging’ studies with available stroke lesion data in recent years. These occurred primarily within the framework of large, international collaborations, such as the Meta VCI Map consortium (∼3000 patients),^27^ ENIGMA (∼2000 patients)^28^ or MRI-Genetics Interface Exploration (MRI-GENIE) (∼2800 patients)^29^ but also in some single centre, or national settings, such as University College London Hospital (∼1300 patients),^5,30^ Hallym University Sacred Heart Hospital or Seoul National University Bundang Hospital (∼1400 patients).^31,32^ In addition, stroke is such a common disease that it may well be feasible to acquire even larger data sets. This aspect is exemplified by ongoing studies, such as the DISCOVERY study with a planned inclusion of 8000 stroke patients.^33^ These increases in data set sizes allow for new opportunities to test their relevance for the performance of deep learning for stroke outcome predictions. At the same time, larger sample sizes for both training and test sets will more reliably protect against biased, i.e. too optimistic estimates of prediction performance that have been observed to occur for prediction studies involving sample sizes up to 150 subjects.^34^

Prediction performance will conceivably increase with data set size independent of the algorithm used.^35^ This means that even linear algorithms are expected to improve up to some asymptotic value. In contrast, deep networks are expected to have a higher asymptotic performance value—since the learnable function space for a deep net is a superset of that of a linear model—at the cost of more challenging optimization. These projections may eventually represent further justifications to invest in costly and time-consuming large-scale study endeavours and motivate deep learning–based approaches.

The present study focuses on the systematic evaluation of deep learning for the prediction of stroke severity based on neuroimaging-derived lesion location information in a large, multicentre cohort.^29^ While there are categorical differences in how linear and deep learning algorithms are trained and optimized—with deep learning being more complex and generally more difficult to optimize^36^—we aimed to develop a methodological setup that represented a fair juxtaposition for both approaches. To get further insights into the role of sample size, we randomly repeatedly subsampled to 3 increasing training data set sizes: 100 patients, 300 patients and 900 patients. Performance was validated in independent patient data. By these means, we aimed to answer the questions: are there non-linear effects between the lesion location and stroke severity that can be leveraged by deep learning models? Do larger stroke data sets comprising ∼1000 patients already represent an advantage over the currently primarily available ones in the range of a few hundred patients?

## Methods

### Patient samples

To increase our sample size, we merged data of patients with acute ischaemic stroke originating from the multicenter MRI-GENIE cohort,^29^ and a retrospective Massachusetts General Hospital (MGH)-based cohort.^37^ We included patients with available quality-controlled DWI-based lesion segmentations and information on acute stroke severity, as measured by the National Institutes of Health Stroke Scale (NIHSS, 0–42: 0, no measured deficits; 42, maximum stroke severity) and obtained during the hospital stay at index stroke. All patients or their proxies of the MRI-GENIE study gave written informed consent in accordance with the Declaration of Helsinki. Given the retrospective character of the MGH-based study, it was performed under a waiver of consent. The study protocols were approved by MGH’s Institutional Review Board (Protocol #: 2001P001186, 2003P000836 and 2013P001024) and the Review Boards of individual sites. The here presented study was conducted in line with the Transparent Reporting of a Multivariable Prediction Model for Individual Prognosis or Diagnosis reporting guideline.^38^

### Neuroimaging data and preprocessing

In this study, we relied on acute MRI-based DWI scans (c.f. Supplementary materials for a detailed description of imaging parameters for the two cohorts). We employed deep learning–based routines for “automatic” DWI-based stroke lesion segmentation in combination with a rigorous manual quality control of each scan. In case of MRI-GENIE, these segmentations were produced by means of a validated ensemble of three-dimensional CNNs.^39^ In case of the MGH-based study, we employed an in-house deep learning–based algorithm (c.f. Supplementary materials for further details).^40^ DWI scans and corresponding lesion segmentations were non-linearly normalized to the common Montreal Neurological Institute (MNI) space.^41^ To ensure a high quality of both lesion segmentations and spatial transformation, we manually evaluated every single spatially normalized DWI scan in combination with the respective lesion segmentation and hence ensured a high quality of the included imaging data [three experienced raters: A.K.B., M.B. (MRI-GENIE) and J.R. (MGH-based study)]. Please note that we hence relied on the qualitative evaluation of automatically generated lesion segmentations by experienced raters, rather than the quantitative comparison of automatically and manually generated lesion segmentations. This decision was motivated severalfold: the critical evaluation of quantitative measures, such as the dice score,^42^ is an essential part of designing segmentation algorithms. However, even a high dice score does not guarantee that a lesion segmentation is flawless. Furthermore, the computation of the dice score requires the creation of ground truths and hence the very time-consuming manual segmentation of stroke lesions, which would not have been feasible given the sizes of employed cohorts. Since the focus of this present work was not the validation of lesion segmentation algorithms but rather the utilization of high-quality imaging data derivatives for stroke outcome prediction, we opted for the thorough, manual evaluation of every single scan. In addition, the three raters were working in close collaboration, with the aim to harmonize the evaluation of individual stroke patients to the maximum extent possible.

Each lesion segmentation comprised binary information for altogether 902 629 voxels, which can conceivably overwhelm prediction algorithms. We, therefore, initially performed a dimensionality reduction step, as commonly done in imaging-based stroke outcome studies.^43,44^ We employed principal component analysis (PCA) of the voxel-wise lesion segmentation information and retained as many components as were necessary for explaining 95% of the variance in the lesion data. Note that this step was completely unsupervised and hence only took the input data (i.e. imaging data) into account but did not have access to the outcome data (i.e. the stroke severity score). Therefore, there was no information leakage between different parts of the data set that could have led to too optimistic performance estimates.

### Computational framework and employed algorithms for the prediction of stroke severity

We repetitively separated the entire data set (total *n* = 1430) into train, validation and test sets of the sizes 915, 229 and 286, respectively.^4^ The test set comprised ∼20% of the entire data set and the validation set ∼20% of the remainder, as is a common convention for small data sets.^45^ We repeated this random split into train, validation and test sets 500 times. In case of the train set, we further subsampled to samples of 100, 300 and 900 patients by drawing from the entire sample of 915 without replacement. We inserted this subsampling step with the idea of getting insights on the effect of sample size, as deep learning algorithms are known to result in better performance for larger data set sizes. We normalized the NIH stroke severity score to be in the range of 0 and 1 by dividing all scores by the maximum of 42. We inserted this preprocessing step to model the outcome as a Bernoulli distribution and utilized a binary cross-entropy (BCE) loss function. We trained algorithms to predict stroke severity via gradient descent using backpropagation relying on Adam optimization.^46^ We opted for a batch size of 64 to enable a fair comparison between training data set sizes and ran as many batches as were necessary to iterate through all examples for an epoch (therefore, 2 for data sets of 100 patients and 14 if 900 patients). To optimize model hyperparameters, we repeated the described training procedure for 49 individual models with different combinations of hyperparameter constellations [learning rate = (1.0, 0.3, 0.1, 0.03, 0.01, 0.003, 0.001), weight decay (regularization): (0.1, 0.03, 0.01, 0.003, 0.001, 0.0003, 0.0001)].

**Figure 1:**
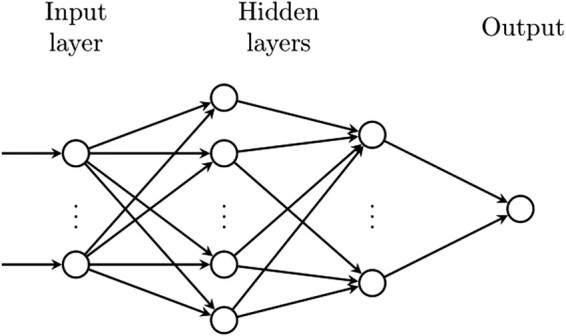
**Graphical scheme of the deep learning model**. Each hidden layer has ReLU^47^ activation functions, and the output has a sigmoid activation function that squashes the output between 0 and 1.

We ran the entire analysis pipeline for two different models that varied in the depth of their architecture. As a baseline model that is also the closest to the ones classically employed in stroke outcome prediction studies,^44^ we implemented a *l_2_*-regularized logistic linear regression model with a sigmoid activation function. This model therefore corresponded to a one-layered neural network. The deepest model that we implemented was an eight-layered neural network with seven hidden layers (dimensions: 512, 256, 128, 64, 32, 16, 8; c.f. Figure 1 for an intuition). While it would in principle be possible to extract the learned parameter settings for the linear regression model, we refrained from doing so given our methodological setup with 500 repetitions and therefore 500 trained models with potentially varying parameter settings.

### Model selection and performance evaluation

We evaluated prediction performance as explained variance based on the coefficient of determination, *R*^2^. We opted for this relative measure of the accuracy of our continuous predictions given its frequent use in previous stroke studies^6,44^ and hence straightforward comparability and its ease of interpretation, given that it relates to the success of the prediction.^48^ We computed the *R*^2^ value in the test set specifically for the model with the overall highest *R*^2^ value in the validation set. To determine this highest *R*^2^ value in the validation set, we selected the checkpoint in the training process that corresponded to the highest *R*^2^ value in the validation set. That is, we trained for a fixed number and then chose the point with the highest *R*^2^ value, rather than stopping training, when the error stopped decreasing. We report the test set *R*^2^ value as averaged across the 500 random splits of the entire data set into train, validation and test sets (c.f. Supplementary Fig. 1 for an overview of our entire analytical pipeline).

### Statistical analysis

Finally, we compared the performance of the two different algorithms with respect to the mean explained variance and linked 95% confidence intervals. We determined significant differences in prediction performance based on non-overlapping confidence intervals.^30,43^

### Sensitivity analyses

To evaluate whether there were any effects of cohort or sociodemographic characteristics, we reran both the linear regression and deep learning model for the largest sample size and estimated the prediction performances for the subgroups: MRI-GENIE cohort, MGH-based cohort, younger patients (≤67.7 years of age), older patients (>67.7 years of age) and male and female patients.

## Results

**Figure 2:**
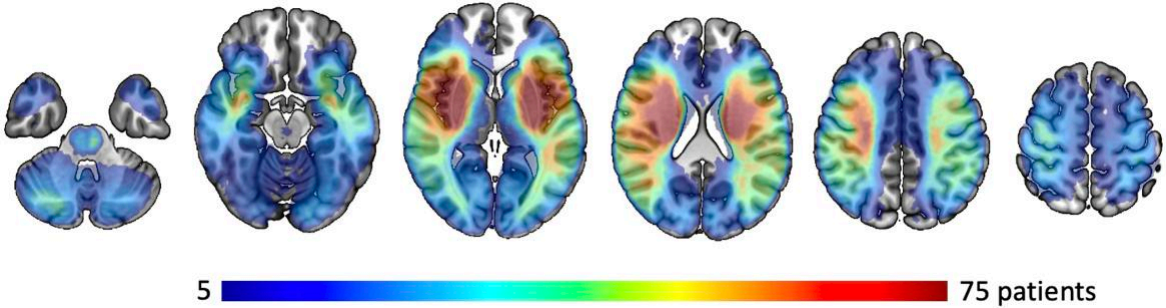
**Lesion overlay of all 1430 patients with acute ischaemic stroke**. The highest lesion overlap was found in middle cerebral artery territory, more specifically subcortically in proximity to the lateral ventricles and insular cortices of both the left and right hemisphere, which was also expected from prior work.^32,49,50^ Fewer lesions affected bilateral territories of the posterior cerebral arteries. Our sample furthermore does not provide sufficient coverage of bilateral anterior cerebral artery territories. Note that this figure presents a heatmap of the lesion frequency for each voxel in the brain; no statistical test was employed.

Our analyses were based on 1430 patients with acute ischaemic stroke (792 MRI-GENIE patients, 638 patients from the MGH-based study; c.f. Supplementary Materials for an overview of the sample size calculations). The average age was 66.3 [standard deviation (SD): 15.0] years, and 43.1% were female patients. Patients had a median acute stroke severity of 4 [interquartile range (IQR): 6]. The median lesion size was 5.0 mL (IQR: 26.7 mL; Table 1; c.f. Supplementary Table 1 for disaggregated, cohort-specific clinical characteristics). Figure 2 presents a lesion overlap visualization (c.f. Supplementary Fig. 2 for a lesion overlap visualization for each of the included cohorts). The highest lesion overlap was located subcortically in middle cerebral artery territory, as well as insular cortex. The PCA dimensionality reduction step resulted in 504 retained components that served as input to our two algorithms.

**Table 1:** Summary of patient characteristics

	Entire sample of patients with acute ischaemic stroke (*n* = 1430)
Age [years, mean (standard deviation)]	66.3 (15.0)
Female sex (%)	43.1
NIHSS-based stroke severity [median (interquartile range)[	4 (6)
Lesion size [mL, median (interquartile range)]	5.0 (26.7)

### Prediction of stroke severity

#### 100–300–900 subjects

**Figure 3:**
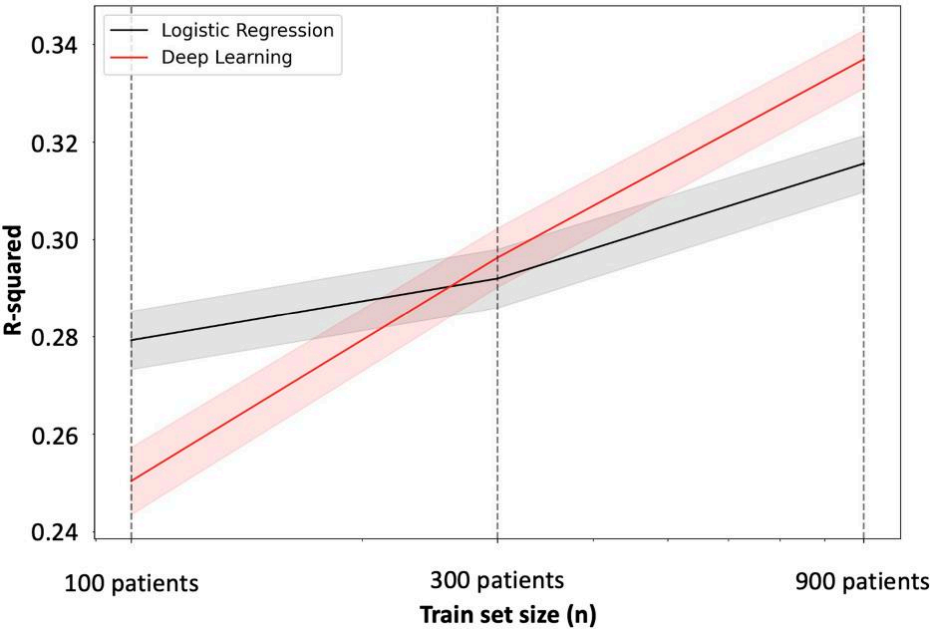
**Average prediction performance of stroke severity on the test set over 500 different random data splits in terms of explained variance (*R*^2^, *y*-axis) depending on train set size (*x*-axis)**. Models were trained for 3 separate train sample sizes of 100, 300 and 900 patients. The *x*-axis here displays those sizes logarithmically. While the linear regression model performed favourably for a sample size of 100 patients, deep learning started to significantly outperform linear regression when trained on 900 patients. Average prediction performance improved by ∼20% when increasing the sample size 9×. This figure presents the mean explained variance (intersection of bold lines and dashed vertical lines for 100, 300 and 900 patients); associated 95% confidence intervals are indicated as shaded areas. No statistical test was employed.

Linear regression resulted in a significantly higher prediction performance compared with deeper models, when training on the stroke data of 100 patients. The mean performances of the linear regression models totalled 0.279 ± 0.005 (*R*^2^, 95% confidence interval), compared with 0.250 ± 0.007 for the deep learning models. The non-overlapping 95% confidence intervals thus showed a significant advantage of the linear method for our smallest tested sample size of 100 patients. In case of a training data set of 300 patients, linear models and deep learning performed similarly well, as indicated by their overlapping 95% confidence intervals: the exact mean performances were 0.292 ± 0.006 for linear regression and 0.296 ± 0.006 for deep learning. The situation of initial superiority was reversed for 900 patients, where deep learning achieved significantly higher prediction performance with an explained variance of 0.337 ± 0.006, compared with the linear model (*R*^2^ = 0.316 ± 0.006; Fig. 3).

Altogether, we thus observed that the maximum performance in mean explained variance when going from 100 to 900 patients increased independent of the employed model: by 0.04 for the linear model and by 0.09 for the deep learning model (Fig. 3).

#### Sensitivity analyses

When rerunning prediction analyses for the largest training sample size (*N* = 900, 50 random initializations) to investigate the effects of cohort, age and biological sex, we obtained the following results: in case of deep learning–based prediction, we estimated the explained variance (*R*^2^) to be 0.36 ± 0.02 for the MGH-based cohort and 0.31 ± 0.03 for MRI-GENIE cohort. The explained variance for female patients was 0.38 ± 0.03, male patients 0.30 ± 0.03, older patients 0.33 ± 0.03 and younger patients 0.36 ± 0.02. Patterns were similar for logistic regression-based prediction: MGH-based cohort, *R*^2^ = 0.34 ± 0.02; MRI-GENIE cohort, *R*^2^ = 0.29 ± 0.03; female patients, *R*^2^ = 0.35 ± 0.02; male patients, *R*^2^ = 0.28 ± 0.02; older patients, *R*^2^ = 0.32 ± 0.03; and younger patients, *R*^2^ = 0.33 ± 0.02.

## Discussion

In this study, we examined the capacity of deep learning for the prediction of stroke severity in relation to linear models and the dependence of training set sample size. Deep learning outperformed more common linear methods for training set sizes of 900 patients with ischaemic stroke. Such an advantage was not detectable for sample sizes of 300 patients, which may be seen as an infliction point, as, in fact, the advantage was reversed for sizes of 100 patients. In this case of small training samples, linear methods performed significantly better than deep learning approaches. Independent from this switchover in best performance, there were notable increases in the prediction performance for both linear and deep methods with larger training set sizes.

Our results match the common notion that deep learning–based prediction performs better for larger sample sizes. However, ‘large’ in the context of deep learning–based prediction studies typically refers to samples with 10^5^–10^7^ examples and not 10^2^–10^3^, as in our study. Thus, it may be a particularly encouraging finding that the benefit of deep learning was already appreciable for our only moderately large sample size. Essentially, the significantly higher performance of deep learning compared with linear models suggests that there are non-linear effects between where in the brain a lesion occurs and the severity of stroke symptoms—effects that may only be captured with more flexible models in combination with a sufficiently high number of observations from which to learn. This existence of non-linear effects may be even less surprising when considering that our outcome variable, the NIHSS score, is a global score and combines impairments in several functional systems at once. Given our findings and the fact that there are increasingly more emerging large-scale stroke imaging data sets comprising data of >10^3^ patients with ischaemic stroke,^27-32^ it may be promising to include more flexible algorithms, such as deep learning, in the collection of routinely employed algorithms to achieve best possible results for outcome prediction. Similarly, our findings indicate that linear models are best used for small data sets, supporting current practice. Furthermore, linear models provide the highest level of transparency^51^ and may hence be the preferred choice of model at any data set size if the goal is interpretability, rather than prediction.

In addition, the evaluation of maximum performance across smaller to larger sample sizes, especially also the gain from 300 to 900 patients, suggests that it is reasonable to expect further gains with yet larger samples than the currently investigated one. In view of their larger increase in performance from smaller to larger samples, this may be especially true for deep learning models. These projections hence support and justify the ambitions of large, international collaborations aiming to recruit several thousand patients with stroke. It remains to be seen, however, whether prediction performances can be increased to an extent that renders them immediately clinically useful. For example, would an increase in prediction performance to an explained variance of 50% be more helpful in clinics than our presented explained variance of 34%, or would clinical utility be given for values of >90% only?

Altogether, several aspects beyond a larger sample size in combination with machine learning techniques and increased prediction performances may influence the clinical utility of future prediction models positively: first, this utility may be directly linked to the importance of the measured outcome scores. Is it an outcome of concrete relevance for patients and their everyday life, such as measure of motor or cognitive impairment? For instance, the Determinants of Incident Stroke Cognitive Outcomes and Vascular Effects on RecoverY (DISCOVERY) study is projected to acquire detailed cognitive scores in 8000 patients with stroke.^33^ Information on the predicted cognitive level of functioning, as derived from this large sample, may be directly helpful to future patients, their families and healthcare professionals, as it will allow for realistic expectations, and optimized tailored care.^52^

Another relevant consideration is the integration of information from additional sources beyond imaging^53^—we here solely considered information on the ischaemic lesion as apparent on the DWI scan that represents, essentially, the location as well as extent of the lesion. Conceivably, the maximum prediction performance will increase with the addition of sociodemographic characteristics and the acute clinical presentation, as well as further imaging-derived information, such as white matter changes,^54,55^ subtle imaging characteristics as captured by radiomics^56,57^ and compound measures, such as the estimated brain reserve.^58^ Once again, it may be particularly important to explore the potential of combining information from several sources in the context of various sample sizes and algorithms. Most likely, the true benefit may only become apparent for larger samples sizes in combination with models that can capture non-linear effects.

### Strengths and limitations

Our study has several strengths. In addition to the availability of a large data set of ischaemic stroke patients with acute imaging, we exclusively present the prediction performance for a test set. This test set was implicated neither in the training of algorithms, which is the estimation of model weights or optimization of hyperparameters, nor their validation. We therefore aimed to avoid data leakage and adhered to established deep learning standards. In this context, it is important to note that while we performed an initial dimensionality reduction step across the entire data set, this step relied on a completely unsupervised technique: the PCA considered imaging data and therefore the input data only. Such an unsupervised step without inclusion of outcome data is generally considered to be valid in a train–test scenario, in contrast to techniques that additionally take information on the outcome into account.^59^

Additionally, we paid great attention to create a fair juxtaposition for the systematic comparison of deep learning and linear models that are typically both linked to varying optimization and training strategies. We here wrote custom code to optimize our linear models in the same train–validation–test split scenario, as optimal for deep learning. Altogether, we ensured that neither the linear nor the deep learning model was disadvantaged.

Further important limitations of our study relate to the lesion segmentation information from clinical DWI scans. Given the acquisition in clinical routine, the resolution of these scans was comparably low. In addition, we used varying automatic lesion segmentation algorithms for each cohort, which could have potentially led to slight differences between the cohorts. In this context, it is important to note that this is a realistic scenario for any study aiming to assemble a data set as large as possible: in most cases, this will require the integration of data originating from different sites, possibly employing different scanners, imaging parameters and imaging processing tools. Hence, it is necessary to conceptualize robust approaches for data harmonization. We here focused on the manual quality control step of spatially normalized lesion segmentations and paid great attention to harmonize our concrete procedures for both cohorts. Resultingly, we expect to have mitigated effects of varying imaging parameters and lesion segmentations on our results to the maximum extent possible. Moreover, in line with previous stroke prediction studies,^6,44^ we employed an initial (linear) PCA step for dimensionality reduction from the image with almost 1 million voxels to 504 principal components. In the future, it may be beneficial to test various linear—and non-linear—approaches for initial dimensionality reduction, as their relevance for prediction performance is currently underexplored. While dimensionality reduction has been an important first step for subsequent training of linear regression algorithms and is commonly performed in stroke outcome studies, deep learning algorithms may be capable of favourably handling voxel-wise data without an intermediate dimensionality reduction step.

Another limitation can be seen in the lack of interpretability of our prediction models, as we focused on maximizing prediction performance while accepting that our methodological pipeline was not optimized for interpretability.^60,61^ In particular, we were specifically interested in designing a fully automatic pipeline that did not rely on any manual or expert-based input. Our aim was to preserve the complexity of the data to a maximum extent possible and, in fact, let the ‘data speak for themselves’.^62^ Future work will be needed to complement our approach and look, exactly, into the derivation of interpretable, relevant lesion patterns.

In sensitivity analyses that were focused on cohort effects and those related to key sociodemographic characteristics, we observed that, independent of the prediction model, prediction performance was estimated to be higher on average for patients of the MGH-based compared with the MRI-GENIE cohort, as well as higher for female compared with male patients. Estimates for younger and older patients were more comparable. While we can therefore describe those patterns in subgroup-specific prediction performance, it was beyond the scope of this study to investigate their explanation. Further studies are warranted to test whether the sex and age effects observed here can be replicated in independent samples and, if so, can be explained by further subgroup-specific characteristics, such as lesion size or lesion location. Lastly, in case of consistent differences in prediction performance per subgroup, it may be of high relevance to develop approaches to mitigate these disparities.^63^

A final limitation is our focus on a global score, such as NIHSS-based stroke severity. NIHSS subscores were not available to us. However, while the NIHSS is a broad compound score, it might be a valid first step to test the capacity of deep learning in the larger sample size regimen. Especially in view of initiatives that introduce recommendations for enhanced data harmonization between different stroke studies,^64^ future studies testing the validity of our conclusions for specific outcomes, such as motor impairments and cognitive functions, may be feasible.

## Conclusion

We here present first evidence that deep learning can predict stroke severity from lesion information significantly better than linear models once the training set size is sufficiently large (900 patients). Conversely, linear models performed significantly better in case of smaller training samples of 100 patients. Prediction performance generally increased with increasing sample size. In summary, our findings suggest the existence of non-linear relationships between lesion location and stroke symptoms that can be captured and utilized to augment the prediction of clinical stroke outcomes based on larger stroke data sets. This increase in prediction performance could then be of unique value for optimizing decisions of acute clinical care and rehabilitation approaches for individual patients.

## Supplementary material

Supplementary material is available at *Brain Communications* online.

## Supplementary Material

fcae007_Supplementary_Data

## Data Availability

The authors agree to make the data available to any researcher for the express purposes of reproducing the here presented results and with the explicit permission for data sharing by individual sites’ institutional review boards. Prediction analyses were implemented in Python 3.7 (predominantly relying on packages: Pytorch 1.9^65^). Jupyter notebooks with exemplary code for reuse are openly available at: https://github.com/bouracha/stroke_outcome_DL_v_LR.
