# Supplementary material for: Scaling behaviours of deep learning and linear algorithms for the prediction of stroke severity
Source: Brain Commun. 2024 Jan 10;6(1):fcae007. doi: 10.1093/braincomms/fcae007 (PMC10808016; doi:10.1093/braincomms/fcae007)
Supplement: fcae007_Supplementary_Data [file fcae007_supplementary_data.docx]

**Supplemental materials**

**Methods**

**Neuroimaging parameters**

**MRI-GENIE:** Neuroimages were obtained in 1T, 1.5T or 3T scanners (General Electric Medical Systems, Philips Medical Systems, Siemens, Toshiba, Marconi Medical Systems, Picker International, Inc.).

**Diffusion-weighted images (DWI):** Mostly axial orientation (2727/2770 axial, 43/2770 coronal). Axial: Reconstruction matrix 256x256mm^2^ (range: 128x128mm^2^ to 432x384mm^2^), median field-of-view 230 mm (range: 200 to 420 mm), median slice thickness 5mm (range: 2 to 7mm, gaps of 0 to 3mm), median TR 4.773ms, median TE 92ms. Coronal: reconstruction matrix 256x256 mm^2^, median field-of-view 260mm, median slice thickness 5mm, median TR 8.200ms, median TE 112ms. Mostly 3 directions (range: 3 to 25). Mostly low b-value 0s/mm^2^ (range: 0 to 50s/mm^2^), high b-value 1000s/mm^2^ (range: 800 to 2000s/mm^2^).

Initially, we automatically created lesion segmentations for a total of 2,765 patients with AIS.^1^ Out of these, 70.1%, i.e., 1,920 segmentations, passed internal quality control for both segmentation quality and spatial normalization quality evaluated by two experienced raters (M.B. and A.K.B). The total sample size was reduced to 1,075 patients after exclusion of all those subjects without any available information on stroke severity and comorbidities. Finally, we additionally excluded all those patients that were recruited at MGH to prevent any overlap in data with the second cohort. The final sample size was 792.

**MGH-based cohort:** Neuroimaging scans were obtained on either a Siemens (Munich, Germany) 3T MRI or a General Electric (Fairfield, CT) 1.5T MRI machine.

**Diffusion-weighted images (DWI):** Echo time: 60 to 120 ms; repetition time: 5300–5600 ms; slice thickness: 5 mm with a 1-mm gap.

A total of 2,120 scans underwent lesion segmentation initially. Out of these scans, 916 were excluded due to being duplicates, late subacute or chronic strokes, hemorrhagic conversion, too much motion degradation, or numerous lesions difficult to segment. The remaining 1,204 scans were spatially normalized to MNI standard space. An additional 297 scans were then excluded due to either insufficient registration quality or difficulties in the registration process, resulting in high quality spatially normalized lesion segmentations for 907 patients. The number of patients included in this study was further reduced to 638 by requiring information on stroke severity.

**Segmentation of stroke lesions**

**MGH-based cohort:** The following preprocessing steps were applied: Data were resampled to $0.89 \text{x }0.89 \text{x} 6$ mm voxel spacing (the median voxel spacing of the training dataset). Data was then skull-stripped, bias corrected and normalized (image intensities with zero mean, unit variance).

We utilized a symmetrical 3D 5-level U-Net architecture^3^ with preprocessed DWI volumes as input and a probability map of the likely stroke lesions as output. Feature map downsampling and upsampling was implemented through strided convolution and trilinear interpolation, respectively. Group Normalization (with group size of 16) in lieu of Batch Normalization is used to accommodate the smaller batch size necessary to train a large patch 3D model. We used rectified linear unit (ReLU) activation in all layers, but the final one, where we used a sigmoid activation function.

We trained our network on patches of size 144x176x24 voxels with batch size 2. Patches were sampled at random during training. The network was trained to maximize the Dice Similarity Coefficient (DSC) between predicted label maps and ground truth, as expressed by the following equation:

$$DSC=1-\frac{2*\sum p*q+\epsilon}{\sum p+\sum q+\epsilon}$$

($p$ is the ground truth, $q$ the predicted map, $\epsilon$ is used to prevent floating point instability when the magnitude of the denominator is small (set to 1)). Training relied on the SGD optimizer with decoupled weight decay, the learning rate was progressively decreased:

$$\eta_{t}=\eta_{min}+0.5*\left( \eta_{max}-\eta_{min} \right)*\left( 1+\cos\left( \frac{\pi T_{curr}}{T} \right) \right)$$

$(\eta_{max}$ initial learning rate (set to 0.1), $\eta_{min}$ final learning rate (set to 0.0001), $T_{curr}$ current iteration counter, and $T$ total number of iterations to train for (set to 150 epochs)).

Furthermore, we applied a weight decay of 0.00002 to all convolutional kernel parameters, leaving biases and scales un-regularized. Real-time data augmentation was employed during the training process (random axis mirror flips (for all 3 axes), isotropic scaling (.75 to 1.25), rotations (-15° to 15°) around all three axes, and gamma corrections (.75 to 1.25), all with probability 0.5).


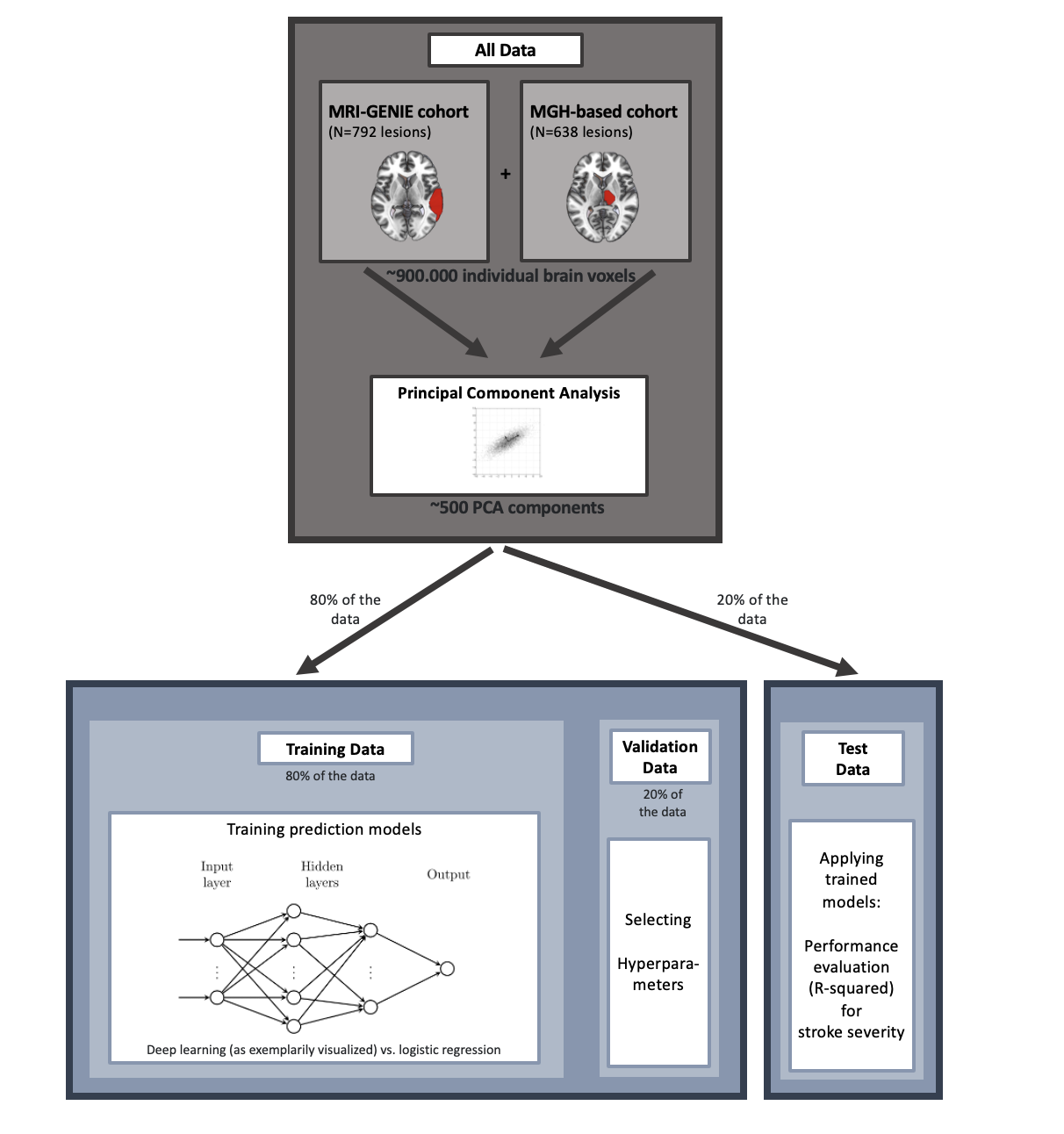


**Supplementary Figure 1. Overview of analytical pipeline.** We first combined individual lesion segmentations that comprised the lesion status of every whole-brain voxel from all patients of our two cohorts (N=1430 lesion segmentations in total). In a next immediate step, we employed principal component analysis (PCA) to reduce the dimensionality of our input space from ~900.000 voxels to 504 PCA components (capturing 95% of the variance of the original input). Afterwards, the entire dataset was split into 80% for training data (80% of the 80%) and validation data (20% of the 80%) and 20% for the test data set, this step was repeated 500 times. We trained our logistic regression and deep learning models in the training dataset, then employed the validation dataset to decide on hyperparameters. The performance of our prediction models was eventually computed in the remaining and unused test data.

**Supplementary Table 1.** **Summary of patient characteristics per included cohort.** Patients of the MGH-based cohort were ~5 years older on average. In contrast to MRI-GENIE, that recruited more male than female patients, there was a balance between male and female patients in the MGH-based cohort. The comparison of average lesion size indicated that patients in the MGH-based cohort had markedly larger lesions, despite comparable NIHSS-based stroke severity scores (same median score of 4, however, the MGH-based cohort had a wider spread of NIHSS scores).

|  | **MRI-GENIE patients with acute ischemic stroke (n=792)** | **MGH patients with acute ischemic stroke (n=638)** |
| --- | --- | --- |
| **Age** (years, mean (standard deviation)) | 63.9 (14.8) | 69.2 (14.7) |
| **Female sex (**%) | 38.3% | 49.1% |
| **NIHSS-based stroke severity** (median (interquartile range)) | 4 (5) | 4 (9) |
| **Lesion size** (ml, median (interquartile range)) | 2.99 (18.2) | 8.38 (36.8) |


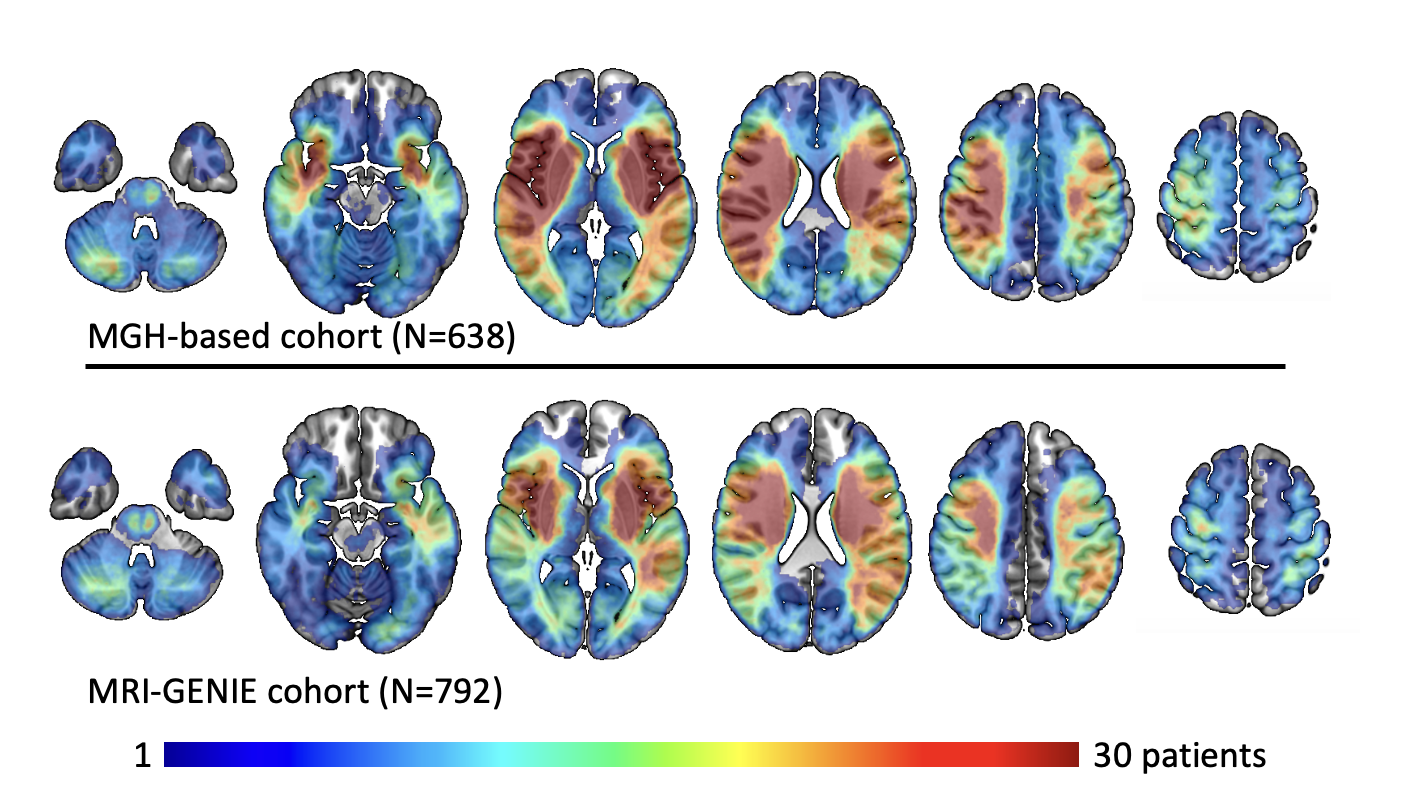


**Supplementary Figure 2. Individual lesion overlay maps for the MGH-based and MRI-GENIE cohorts.** Despite differences in median lesion volume (c.f., **Supplementary Table 1**), both cohorts showed qualitatively similar lesion distributions, with the highest lesion overlap being located subcortically in bilateral MCA territories.

**Supplementary References**

1. Wu O, Winzeck S, Giese AK, et al. Big Data Approaches to Phenotyping Acute Ischemic Stroke Using Automated Lesion Segmentation of Multi-Center Magnetic Resonance Imaging Data. *Stroke*. Published online 2019:STROKEAHA. 119.025373.

2. Bonkhoff AK, Schirmer MD, Bretzner M, et al. Outcome after acute ischemic stroke is linked to sex-specific lesion patterns. *Nat Commun*. 2021;12(1):3289. doi:10.1038/s41467-021-23492-3

3. Chang K, Brown J, Beers A, Rosen B, Kalpathy-Cramer J, Ay H. Abstract WMP17: Fully-Automated Ischemic Brain Infarct Volumetric Segmentation in Diffusion Weighted MR using Deep Learning. *Stroke*. 2019;50(Suppl_1):AWMP17-AWMP17.
